# Co-developing equity-oriented neighbourhood care for children and young people with multiple long-term conditions: a concept mapping study in South London

**DOI:** 10.1093/eurpub/ckag187

**Published:** 2026-10-15

**Authors:** Nandeeta Samad, Ingrid Wolfe

**Affiliations:** Department of Women & Children’s Health, King’s College London, London, United Kingdom; Department of Women & Children’s Health, King’s College London, London, United Kingdom; CHILDS Programme, Evelina London, Guy’s and St Thomas’ NHS Foundation Trust, London, United Kingdom

## Abstract

Children and young people (CYP) with multiple long-term conditions (MLTCs) rely on services spanning health, education, and social care, but fragmented pathways may widen inequalities and shift coordination work to families. We aimed to identify priorities for equity-oriented neighbourhood care. We conducted a participatory mixed-methods study informed by the Medical Research Council framework and group concept mapping. Sixteen stakeholders participated: three CYP, seven parents, five healthcare professionals (HCPs), and one community stakeholder. Participants generated, sorted, and rated candidate components. Group data were analysed separately using multidimensional scaling and hierarchical cluster analysis, then synthesised through stakeholder interpretation and consensus. The Rainbow Model of Integrated Care informed interpretation. HCPs and community stakeholders refined 38 candidate neighbourhood-care statements to 27 and generated five clusters; families refined 52 candidate neighbourhood-care statements to 36 and generated four clusters. Seven priorities were co-developed: tailored information, local access, and pathway communication; named coordination; workforce capability; shared care-planning tools; multidisciplinary working; parent/carer support; and embedded mental health support. Communication, local access, and navigation were relatively feasible, whereas mental health and cross-sector integration were resource-dependent. A stakeholder-informed programme theory linked components to accountability, information-sharing, culturally responsive care, earlier escalation, and reduced family burden. The findings translate neighbourhood-care policy into co-developed, equity-oriented priorities for CYP with MLTCs in South London. Communication, local access, and coordination offer practical starting points, while mental health and cross-sector integration require phased resourcing and leadership. These priorities may inform adaptation in European child health systems.

## Introduction

Multiple long-term conditions (MLTCs) in childhood are an emerging public health challenge because they intersect with physical and mental health, development, education, family life, and social disadvantage. In England, an estimated 125 159 children and young people (CYP) aged 0–19 years were living with MLTCs in 2024, with a higher burden in more deprived groups and variation by ethnic background [1]. Adult multimorbidity models do not fully capture growth, family dependence, education, or transition through adolescence, so integrated care for CYP must address these developmental and cross-sector needs [2].

Integrated care may improve quality of life and care quality for CYP with ongoing conditions, but effects on emergency care use and costs remain mixed [2, 3]. South London families have valued joined-up care attentive to wider child and family needs [4]. The Children and Young People’s Health Partnership (CYPHP) trial did not reduce unplanned admissions and was not cost-effective at 6 or 12 months, although eczema symptoms and asthma care improved [5, 6]. Its process evaluation identified variable implementation and low reach related to resources, competing priorities, and change over time, which may have limited effects on service use and child health outcomes [7].

The NHS 10 Year Health Plan prioritises neighbourhood multidisciplinary care for CYP with complex health and social care needs and involvement of CYP and families in its development [8]. Guidance on complex interventions similarly recommends early attention to context and implementation [9].

Previous South London research provided the empirical basis for this study. A qualitative health needs assessment involving 24 interviews identified poor coordination, limited continuity, care that did not fit family life, and barriers related to poverty, language, digital access, and trust (Samad N, Oluyase AO, Pickard L, Wolfe I, unpublished observations, 2026). Earlier local assessment found high levels of unmet physical, mental, and social needs [10]. Recent national linked-data work further characterised the incidence and prevalence of multimorbidity among CYP in England (Aveline A, Ward J, Tydeman F, Swann O, Wolfe I, Fraser LK, unpublished observations, 2026). Children in the most socioeconomically deprived area-level quintile, and Asian and Black children compared with White children, had lower odds of engaging with case-finding; once engaged, those in the most deprived quintile were more likely to require further or specialist care [11]. These findings indicate that intervention development must address equity as well as integration.

Previous work identifies unmet need, inequalities, and implementation challenges [5–7, 10, 11], but not which neighbourhood-care components stakeholders consider important and feasible, how priorities differ, or how components link to programme theory. We address this gap using group concept mapping to prioritise theory-informed components for testing.

Co-development can support intervention design by involving those who receive and deliver care, although co-design terminology and reporting remain inconsistent [12]; CYP involvement may also become tokenistic without careful planning [13]. We therefore aimed to co-develop priorities for an equity-oriented neighbourhood-care model for CYP with MLTCs in South London. The objectives were to identify modifiable targets, reach consensus on important and feasible components, and develop a prioritised framework for future testing. Although health-system arrangements differ, coordinating care across primary, specialist, community, mental health, education, and social services without widening inequalities is relevant to diverse European urban health systems.

## Methods

### Study design

This participatory mixed-methods intervention development study was informed by the Medical Research Council (MRC) framework for complex interventions [9]. It focused on context, programme theory, stakeholder priorities, feasibility, and future delivery. The Rainbow Model of Integrated Care (RMIC) was used to interpret how components related to clinical, professional, organisational, functional, system, and normative integration [14].

We followed Trochim’s group concept mapping approach [15, 16]. Concept mapping was selected as a method because it combines qualitative idea generation with quantitative sorting and rating. It also produces visual maps that show how stakeholders understand relationships between intervention ideas. This supported MRC-informed intervention development by structuring candidate components and assessing their importance, feasibility, and contextual fit.

### Stage 1: Preparation and stakeholder mapping

The study was conducted from 1 January to the end of June 2026. Stakeholders were recruited through Guy’s and St Thomas’ NHS Foundation Trust and King’s College Hospital NHS Foundation Trust. Recruitment used purposive stakeholder sampling to include people with lived, clinical, or implementation knowledge relevant to neighbourhood care.

Stakeholders were identified using World Health Organization (WHO)-informed stakeholder mapping principles [17]. Consistent with the MRC focus on context and stakeholder engagement, we mapped stakeholders by their relationship to the proposed intervention, likely influence on implementation, lived or professional experience, and relevance to neighbourhood care. This helped include people affected by the intervention, people likely to deliver it, and people with knowledge of the local care system.

Sixteen stakeholders participated in two groups: three CYP and seven parents in the family group, and five healthcare professionals (HCPs) plus one community stakeholder (a family support worker), in the HCPs and Community stakeholders group (Supplementary Table S1).

The study was conducted in accordance with the Declaration of Helsinki. Ethical approval was obtained from the NHS Health Research Authority and Health and Care Research Wales (REC reference 25/PR/1505; IRAS ID 356551). All adult stakeholders and participating CYP provided written informed consent; parent consent was obtained where required under the approved protocol.

### Stage 2: Generating and refining statements

Ideas were generated through two group-specific engagement activities, one with the family group and one with HCPs and community stakeholders. All 16 stakeholders responded to the single focus prompt:

*“An integrated, equity-enhancing intervention for children and young people with multiple long-term conditions in South London should include…”*

The prompt captured clinical, practical, family, school, community, and system-level components while remaining focused on the proposed intervention [9].

The research team removed duplicates, combined overlapping ideas, and simplified wording while retaining meaning. Six stakeholders, three family stakeholders (tow parents and one CYP) and three HCPs, reviewed the refined statements for clarity and suitability. This produced separate statement sets for HCPs and community stakeholders and for the family group.

### Stage 3: Structuring the statements

All 16 stakeholders independently sorted and rated the refined statements using Microsoft Word worksheets returned through document-sharing. These individual tasks were conducted separately by stakeholder group [15, 16].

For sorting, stakeholders grouped the refined statements into clusters that made sense to them. They followed three rules: each statement could be placed in one cluster only; single-statement clusters were avoided unless unavoidable; and all statements could not be placed in one broad cluster. They also provided a short name for each cluster.

Each statement was rated on five-point importance and feasibility scales from 1 (not important/not feasible) to 5 (very important/very feasible). This established format [15, 16] balances discrimination and task burden. Feasibility reflected perceived deliverability in the local neighbourhood context.

### Stage 4: Data analysis

After statements had been sorted and rated, the resulting data were entered and analysed using the RCMap package in R [18]. Data from HCPs and community stakeholders and from the family group were analysed separately before the results were compared and interpreted together.

Sorting data were converted into binary co-occurrence matrices. If a stakeholder placed two statements in the same cluster, the relevant cell was coded as 1. If the statements were not placed together, the cell was coded as 0. Individual matrices were then summed across stakeholders to create a similarity matrix, showing how often each pair of statements was sorted together.

Multidimensional scaling was applied to the similarity data to generate two-dimensional point maps, following established concept mapping procedures [15, 16]. Statements placed closer together on the map were those more often sorted together by stakeholders. The consistency of the maps was assessed using split-half reliability, with a correlation of 0.70 or above considered acceptable.

Hierarchical cluster analysis grouped statements into non-overlapping clusters. Ward.D2 was used because its minimum-variance criterion favours compact, internally coherent groups [19]. Final solutions considered statistical fit, interpretability, and stakeholder sense-making. Average silhouette scores above 0.50 indicated acceptable cluster structure [20].

In group concept mapping, multidimensional scaling and hierarchical cluster analysis use aggregated statement-by-statement similarity matrices rather than participant-level observations [15, 16]. Sorting data supported separate maps for families (*n* = 10) and HCPs and community stakeholders (*n* = 6). Given small samples, maps were treated as exploratory rather than population estimates. Separate analysis was necessary because groups structured different statement sets; split-half reliability and silhouette scores assessed map consistency.

Mean importance and feasibility ratings were calculated for statements and clusters. Go-zone maps identified statements above both group means, which were treated as high-priority candidate components. Kendall’s tau assessed the association between importance and feasibility ratings within each stakeholder group.

### Stage 5: Interpretation of maps

All 16 stakeholders reviewed their group’s cluster and go-zone maps and ratings, discussed whether groupings were meaningful, considered labels, and identified statements for the intervention. These judgements informed cluster interpretation before cross-group consensus (Supplementary Table S2).

### Stage 6: Utilisation of the maps

All 16 stakeholders completed a structured document-mediated consensus activity. They reviewed a document comparing both groups’ maps, candidate components and proposed priorities, and recorded agreement or suggested revisions. Final priorities reflected areas of agreement, while differences in emphasis were retained in the interpretation. High-importance, lower-feasibility statements were retained where they addressed equity.

Following the consensus activity, the research team constructed a stakeholder-informed programme theory by mapping the agreed priorities and candidate components to illustrative delivery activities, proposed mechanisms, contextual conditions, and anticipated outcomes. The model was informed by the Rainbow Model of Integrated Care [14] and the preceding needs assessment. Its mechanisms and outcomes were not empirically tested in this study.

Nine engagement activities were completed: two idea generation, two statement review, two structuring, two map interpretation, and one consensus activity (Supplementary Table S2).

## Results

The cluster maps show how stakeholders organised related statements, while go-zone maps identify candidate components rated above the group means for both importance and feasibility.

### Concept mapping with HCPs and community stakeholders

HCPs and community stakeholders refined 38 initial statements into 27. Split-half reliability was 1.00. A five-cluster solution had an average silhouette score of 0.70 (Fig. 1A; Supplementary Table S3): Accessible and Responsive Care, Clinical and Professional Integration, Community and System Links, Functional Integration, and Whole-Child and Family Support.

**Figure 1:**
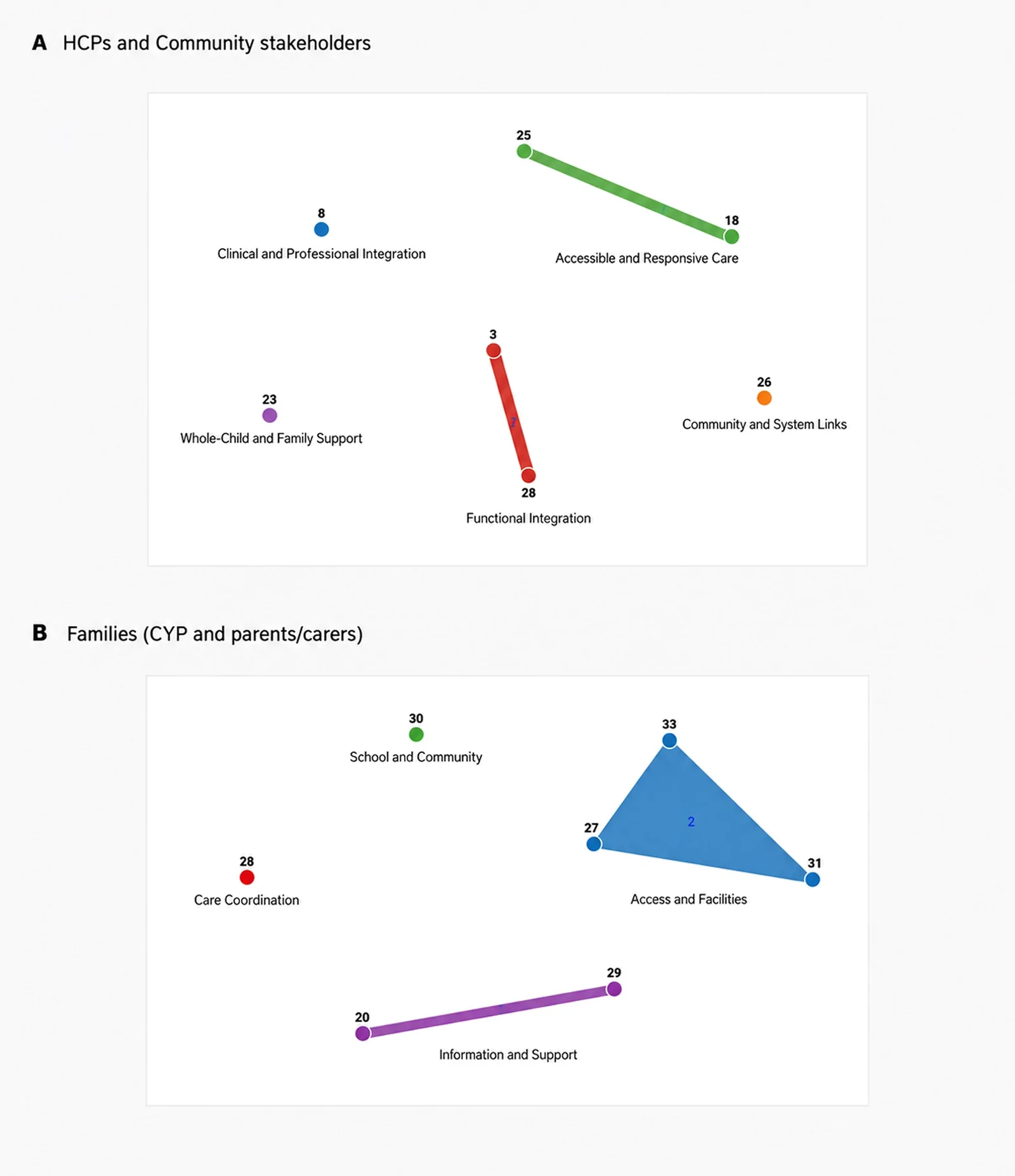
Cluster maps for (A) HCPs and community stakeholders and (B) families (children and young people and parents). Statements positioned closer together were sorted together more frequently.

Across the five clusters, mean importance ranged from 4.50 to 4.83 and feasibility from 3.08 to 3.83 (Supplementary Table S3), indicating high importance but greater variation in perceived deliverability.

Accessible and Responsive Care ranked highest when importance and feasibility were considered together. Its eight statements covered neighbourhood multidisciplinary working, local settings, school-based clinics, longer or specialist general practice (GP) appointments, staff training, whole-child assessment, reasonable adjustments, and family context. Mean importance was 4.62 and feasibility 3.83.

Clinical and Professional Integration ranked second, covering named link roles, follow-up responsibility, escalation, multidisciplinary review, cross-sector discussion, and medication review (importance 4.71; feasibility 3.67), indicating high value but dependence on workforce and pathway capacity.

Community and System Links scored 4.50 for importance and 3.72 for feasibility, while Functional Integration scored 4.50 and 3.60, reflecting support for shared documentation, care plans, action logs, and system links, but with practical infrastructure constraints.

Whole-Child and Family Support had the highest importance (4.83) but lowest feasibility (3.08), suggesting that emotional wellbeing and family support were valued but difficult to deliver within current capacity.

### Go-zone map for HCPs and community stakeholders

The go-zone map for HCPs and community stakeholders is shown in Fig. 2A. Mean cut-offs were 3.62 for feasibility and 4.64 for importance; statements 1–3, 12, 14–18, and 25 were in the upper-right quadrant. Importance and feasibility ratings were positively associated (Kendall’s tau = 0.36, *P* = .029).

**Figure 2:**
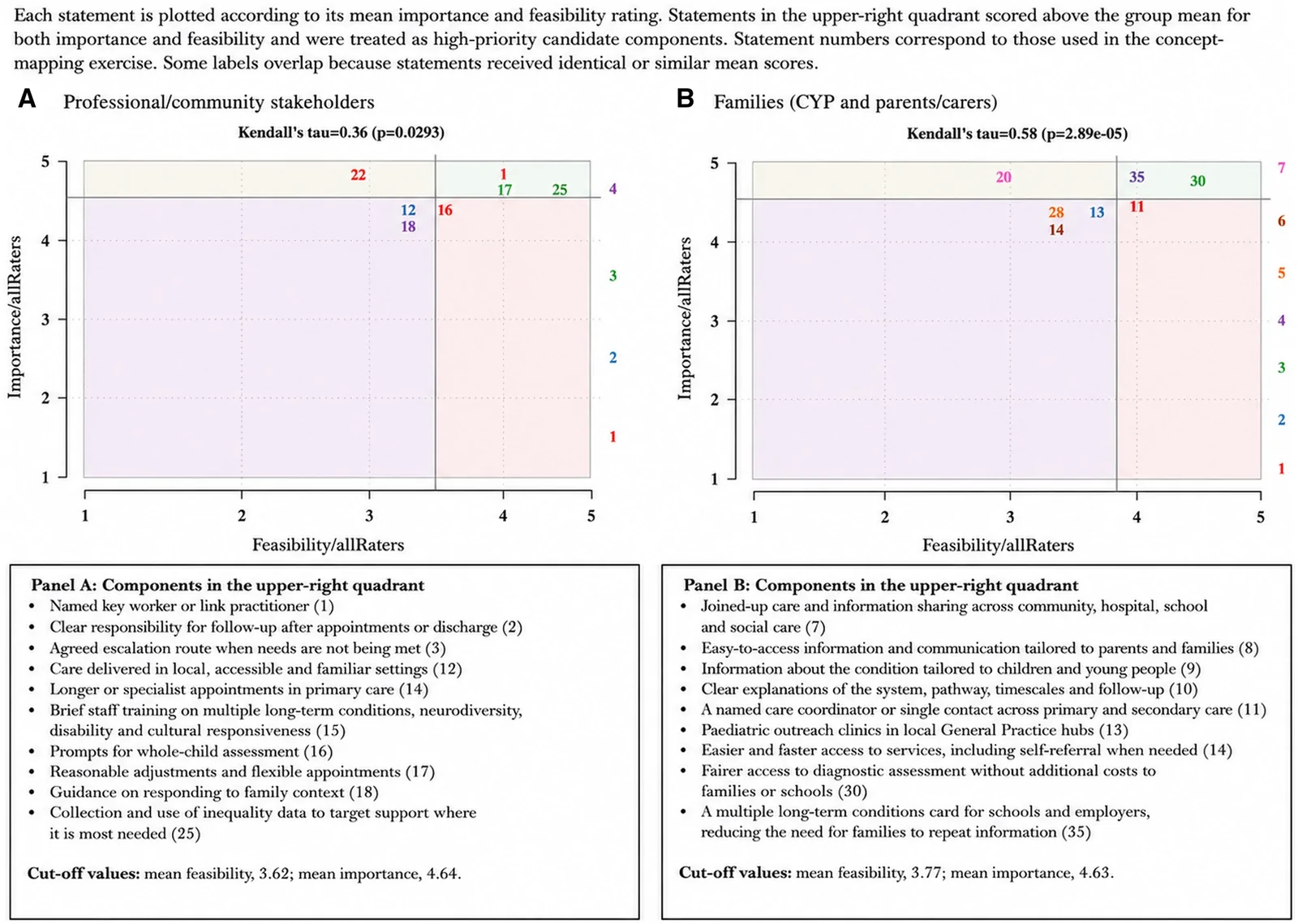
Go-zone maps for (A) HCPs and community stakeholders and (B) families. Statements in the upper-right quadrant were rated above the group mean for both importance and feasibility. Kendall’s tau shows the association between importance and feasibility ratings.

Go-zone findings informed candidate components including named coordination, follow-up responsibility, escalation, staff capability, reasonable adjustments, shared care tools, and multidisciplinary review. Important but less feasible statements were retained where they would require additional resources, pathway redesign, or cross-service agreement.

### Concept mapping with families

In the family group, 52 initial statements were refined into 36. Split-half reliability was 1.00. A four-cluster solution had an average silhouette score of 0.90 (Fig. 1B; Supplementary Table S4): Care Coordination, Access and Facilities, Information and Support, and School and Community.

Across the four family clusters, mean importance ranged from 4.48 to 4.67 and feasibility from 3.48 to 3.92 (Supplementary Table S4), indicating high importance but variable deliverability.

Access and Facilities ranked highest overall. Its 12 statements covered staffing and overnight support during admissions, accessible facilities, local GP outreach, family-initiated referral, urgent specialist support, interim care packages, flexible appointments, cost-free diagnostics, teenager-friendly spaces, accessible equipment, and blended diets. Mean importance was 4.66 and feasibility 3.92.

Information and Support had the highest importance (4.67) and feasibility of 3.73. It covered translated information, pathway maps, reminders, culturally tailored resources, parent training, support while waiting, appointment preparation, child voice prompts, mental health access, and support for single parents.

Care Coordination scored 4.61 for importance and 3.76 for feasibility, reflecting the value placed on joined-up information, single contact routes, hospital passports, family-held summaries, assessment-panel evidence routes, and transition support.

School and Community scored lowest across both variables (4.48 importance; 3.48 feasibility), suggesting that wider school, community, and representation components were valued but seen as more dependent on cross-sector capacity and partnership working.

### Family go-zone map

The family go-zone map is shown in Fig. 2B. Mean cut-offs were 3.77 for feasibility and 4.63 for importance; statements 7–11, 13, 14, 30, and 35 were in the upper-right quadrant. Importance and feasibility ratings were positively associated (Kendall’s tau = 0.58, *P* < .001).

The go-zone map identified candidate components including accessible facilities, flexible appointments, local outreach, translated information, pathway maps, reminders, single contact routes, hospital passports, and family-held summaries. Important but less feasible needs were retained where implementation would require additional staffing, infrastructure, or cross-sector arrangements.

### Convergence and differences between stakeholder groups

Both stakeholder maps converged on clearer coordination, better information-sharing, responsive care, and support for families navigating multiple services. Shared priorities included named coordination, single contact routes, hospital passports or care plans, documentation of agreed actions, and clearer escalation.

HCPs and community stakeholders emphasised professional and functional integration, including multidisciplinary review, defined responsibilities, shared documentation, and medication review. Families emphasised service usability, including staffing during admissions, accessible facilities and equipment, local outreach, flexible appointments, and reduced financial or administrative barriers. These differences were complementary, indicating a need for both professional integration and accessible, family-centred design.

### Co-developed priorities and candidate components

The cluster and go-zone findings were synthesised into seven co-developed priorities and candidate components for neighbourhood care (Table 1). Priority-level means summarise selected components, rather than all statements within each broader cluster.

**Table 1:** Co-developed intervention priorities and candidate components for neighbourhood care for CYP with MLTCs in South London.^a^

Priority	Candidate components	RMIC domains	Mean importance	Mean feasibility
Tailored multilingual information, local access and pathway communication	Translated condition information and clinic letters; simple pathway maps and follow-up prompts; culturally tailored print, video, text, or WhatsApp information; local outreach in General Practice hubs; clearer referral routes; inequality data to target access support	Normative; Functional; Organisational	4.90	4.40
Named coordination roles	Named key worker or link practitioner; clear responsibility for follow-up; single contact route; agreed escalation route when needs are not being met	Clinical; Professional	5.00	4.00
Workforce capability and responsive care	Brief training on MLTCs, neurodiversity, disability, and cultural responsiveness; whole-child assessment prompts; reasonable adjustments; flexible appointments; guidance on family context	Professional; Normative	4.67	4.00
Shared care-planning and communication tools	Shared care plan, hospital passport or MLTC card; standardised letters; family-held summary; clear documentation of agreed actions; and information-sharing across settings	Functional; Clinical	4.50	3.68
Multidisciplinary review and joint working	Regular case review; joint discussion across paediatrics, primary care, mental health, school, and social care; shared action log; clear review; and escalation arrangements	Professional; Organisational; Clinical	4.50	3.33
Parent and carer support, training, and navigation	Training on condition management and service navigation; coaching while waiting; community and peer support; appointment preparation; and follow-up	Clinical; Normative	4.30	3.30
Embedded mental health support	Routine emotional wellbeing check-ins; brief support within paediatric or neighbourhood pathways; warm handover or referral; support while waiting	Clinical; System	5.00	3.00

a Priorities are ordered broadly by feasibility among high-importance priorities. Means are priority-level scores for selected candidate components, not cluster means. RMIC, Rainbow Model of Integrated Care.

The priorities were organised using the RMIC to show how the proposed model addresses connected forms of integration. Importance scores ranged from 4.30 to 5.00 and feasibility scores from 3.00 to 4.40.

The most feasible priority was tailored multilingual information, local access and pathway communication (importance 4.90; feasibility 4.40), spanning normative, functional and organisational integration. Embedded mental health support had joint-highest importance (5.00) but lowest feasibility (3.00), indicating a gap between recognised need and system capacity.

Clinical and professional integration components, such as named coordination and workforce capability, were highly rated and moderately feasible, suggesting scope for integration into existing roles. Organisational or system components, including multidisciplinary review, parent/carer support, and mental health pathways, were less feasible and may require phased development and additional capacity.

### Programme theory and mechanisms of change

The stakeholder-informed programme theory (Fig. 3) links the seven priorities to illustrative delivery activities, proposed mechanisms, anticipated outcomes, and contextual conditions. Neighbourhood infrastructure, named roles, shared tools, inequality data, and community links create conditions for delivery. Activities include translated information, local access and supported referral routes, targeted support, care-planning, staff prompts, multidisciplinary review, and mental health handover. Proposed mechanisms include clearer ownership, better information-sharing, culturally responsive care, earlier escalation, support while waiting, and greater parent confidence.

**Figure 3:**
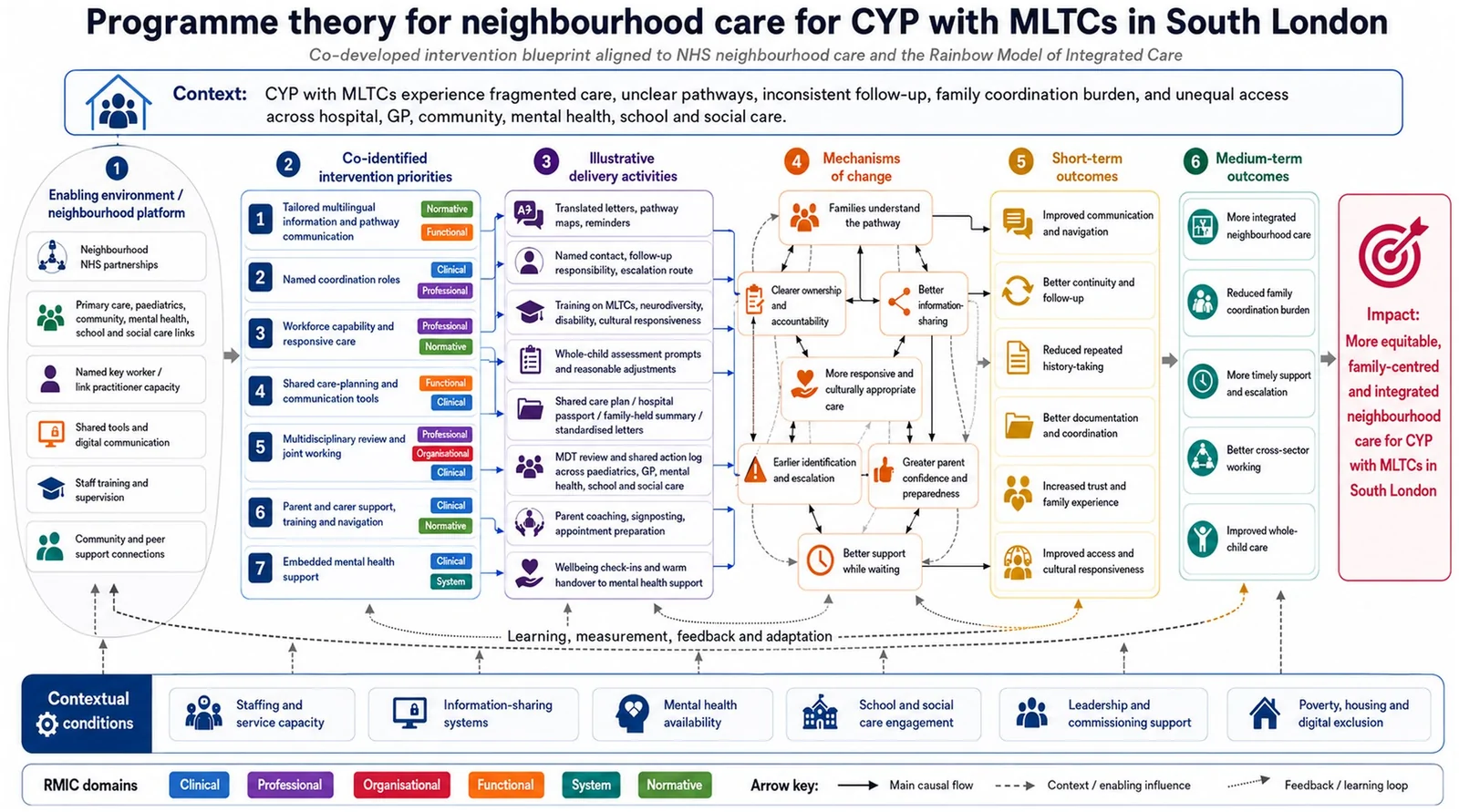
Stakeholder-informed programme theory linking co-developed priorities to illustrative delivery activities, proposed mechanisms, anticipated outcomes, and intended impact for neighbourhood care for children and young people with multiple long-term conditions.

This interpretation fits the RMIC pattern in the table. The most feasible components sit mainly within normative and functional integration. They may be early wins because they improve communication, trust, navigation, and access without major service redesign. Components linked to clinical and professional integration, such as coordination roles and workforce capability, appear deliverable if embedded into existing roles and workflows. The main challenge is at organisational and system levels. Multidisciplinary review, parent/carer support at scale, and embedded mental health support are highly valued, but depend on protected time, workforce capacity, thresholds, and cross-sector commitment.

The model identifies practical starting points while showing that deeper organisational and system integration requires phased resourcing, leadership, and learning.

## Discussion

Stakeholders prioritised functions that make care easier to use, coordinate, and access fairly. The public health contribution is a prioritised account of what equity-oriented neighbourhood care should do and which components may be feasible first.

Families of CYP with MLTCs often become default care coordinators [21–23]. Family-centred care should respond to family life [22], but our findings show that usability also determines whether integration works in practice. Burden-of-treatment and minimally disruptive medicine literature suggests that care can become ineffective when its workload exceeds family capacity [24, 25]. Integration should therefore be judged partly by whether it reduces the work families must do to make services function.

The findings also support a cautious view of integrated care. Some intensive paediatric models have improved outcomes when coordination was well resourced and linked to specialist support [26]. Yet effects across integrated care programmes remain mixed. This is often because coordination is treated as a light-touch role, not as a funded delivery function. Implementation science helps explain this. Complex interventions depend on context, workforce capacity, relationships, information flow, and authority to act across organisational boundaries [9, 27, 28]. For CYP with MLTCs, this is especially important because care crosses health, mental health, education, social care, and family life. This is also where the RMIC interpretation is useful: it shows that integration depends on practical tools and shared values, but also on organisational capacity, system pathways, and authority to act [14].

This study translates current English policy into practical priorities. The NHS 10 Year Health Plan promotes neighbourhood services, care closer to home, shared planning, records, and digital access [8]. NICE guidance recommends early transition planning, joint working, named coordination, and care that reduces treatment burden [29, 30]. The findings indicate that neighbourhood care should be accountable, family-facing, and equity-oriented, not only locally organised. More broadly, they address public health challenges relevant to diverse European urban systems, including cross-sector coordination, culturally and linguistically accessible communication, mental health integration, and family care burden.

The equity contribution is central. Fragmentation of care is not experienced equally. Evidence shows that language barriers can affect healthcare access and quality, and that professional interpretation can improve care [31]. Evidence also links racism with poorer health and healthcare experiences for CYP [32, 33]. UK evidence links poverty, poor housing, and digital exclusion with worse child health and reduced access to care [34–37]. Equity therefore cannot be added later as a minor adaptation. It has to be built into the intervention logic from the start. This strengthens the NHS aim to reduce inequalities and aligns with people-centred care [8, 38].

Neighbourhood care will not reduce fragmentation if responsibility remains unclear or families continue bridging service gaps. Commissioners should fund coordination, shared planning, school links, mental health input, and accessible communication as core functions [8]. Digital routes also require accessible alternatives [37]. Some components may begin through better communication, navigation, and coordination, while deeper organisational and system integration will require phased investment, leadership, and workforce capacity.

By linking the prioritised components to proposed mechanisms and anticipated outcomes, the programme theory makes intervention assumptions explicit and provides a testable framework for future feasibility and evaluation studies. The next phase should define core functions, delivery modes, provider roles, materials, and adaptation processes [9]. Evaluation should examine acceptability, feasibility, adoption, fidelity, reach, and equity alongside child and family outcomes [27, 28].

### Strengths and limitations

A key strength is that intervention development was grounded in local need, patient and public involvement, and multi-stakeholder judgement. The preceding qualitative and local quantitative needs assessments strengthened coherence by anchoring co-development in known gaps in access, coordination, and family workload. The local focus also supports contextual fit for future testing.

Limitations include the 16-person sample and limited direct CYP participation. Three CYP participated in all concept-mapping stages, but as three of 10 family stakeholders analysed with parents, family priorities may more strongly reflect parent perspectives and cannot isolate CYP-specific priorities. We therefore interpret their data as family-informed rather than standalone CYP views; future work should recruit a larger, more diverse CYP sample and analyse their priorities separately. Feasibility ratings reflect current conditions, and programme theory mechanisms and outcomes remain hypotheses for future evaluation.

## Conclusion

These findings provide a prioritised, theory-informed framework rather than a fully specified intervention. Neighbourhood care for CYP with MLTCs should be judged by whether it improves access and continuity and reduces families’ coordination work. Future testing should assess reach and equity alongside feasibility, adoption, and child and family outcomes.

## Supplementary Material

ckag187_Supplementary_Data

## Data Availability

The datasets generated and analyzed during the current study are available from the corresponding author on reasonable request.

## Use of generative artificial intelligence

During article preparation, the authors used ChatGPT (OpenAI) to support language editing, structural refinement, and formatting for journal submission. The authors reviewed and revised all outputs, verified the scientific content and references, and take full responsibility for the final article.

Key points

- Families, HCPs, and Community stakeholders prioritised accessible communication, named coordination, and locally responsive care for children and young people with multiple long-term conditions.
- Communication, local access, and navigation were considered more feasible than mental health and cross-sector integration.
- Integrated care should be judged by whether it reduces unequal access and the coordination work transferred to families.
- Commissioners should fund coordination, shared planning, and accessible communication as core neighbourhood-care functions.
