# Supplementary material for: Co-developing equity-oriented neighbourhood care for children and young people with multiple long-term conditions: a concept mapping study in South London
Source: Eur J Public Health. 2026 Oct 15;36(6):ckag187. doi: 10.1093/eurpub/ckag187 (PMC13671751; doi:10.1093/eurpub/ckag187)
Supplement: Supplementary file 1 [file ckag187_supplementary_data.docx]

**Supplementary file**

**Table 1:** Stakeholder mapping grid

| **NHS/neighbourhood care level** | **Organisation/setting** | **Stakeholder group** | **Level of influence** | **Type of influence** | **Priority of engagement** | **Planned role and type of engagement** |
| --- | --- | --- | --- | --- | --- | --- |
| **Children, young people and family level** | Homes, community settings and care pathways across primary, community and hospital care | **Children and young people with multiple long-term conditions** | High | Primary beneficiaries of the proposed neighbourhood care model; provide lived experience of fragmented care, repeated appointments, school impact, communication barriers and unmet support needs | High: inform, involve, collaborate | Share experiences of accessing care, identify what matters to them, rate the importance and feasibility of candidate components, and help interpret whether proposed priorities are acceptable and meaningful |
| **Children, young people and family level** | Homes, community settings and care pathways across primary, community and hospital care | **Parents and carers of children and young people with multiple long-term conditions** | High | Carry day-to-day care coordination, appointment management, advocacy, communication across services and support while children are waiting for care | High: inform, involve, collaborate | Generate and refine intervention ideas, assess feasibility from a family perspective, identify barriers to access, communication and equity, and support interpretation of priorities for neighbourhood care |
| **Primary care and neighbourhood clinical level** | General Practice, Primary Care Networks and neighbourhood care pathways | **General Practitioners** | High | Influence referral, diagnosis, continuity, follow-up, access to local care, escalation and coordination between primary and secondary care | High: inform, involve, collaborate | Advise on deliverability within General Practice and neighbourhood workflows, clarify feasible coordination and referral routes, and assess how candidate components could be embedded into routine care |
| **Primary care and neighbourhood clinical level** | General Practice, community nursing and neighbourhood care pathways | **Nurse** | Moderate to high | Influences day-to-day clinical support, care planning, monitoring, patient education, family communication and continuity of care | Important: inform, involve, collaborate | Provide clinical and practical insight into follow-up, reasonable adjustments, family education, shared care planning and feasible delivery of support within existing roles |
| **Medicines and primary care support level** | General Practice, community pharmacy or neighbourhood medicines support | **Pharmacist** | Moderate to high | Influences medicines management, medication review, adherence support, safety advice, communication about medicines and links between families and clinical teams | Important: inform, involve | Advise on medicines-related needs, family-held information, medication communication, follow-up prompts and the feasibility of pharmacist involvement in neighbourhood support |
| **Family support and navigation level** | Family support services, community-facing support and neighbourhood care pathways | **Family support worker** | Moderate to high | Influences navigation, practical support, signposting, communication with families, support while waiting and links to community or peer resources | Important: inform, involve, collaborate | Advise on family navigation needs, practical support, culturally responsive communication, appointment preparation, signposting and support for families who face barriers to accessing care |

**Table 2:** Stakeholder engagement activities

| **Concept mapping stage** | **Engagement activity** | **Stakeholders involved** | **Number of activities** |
| --- | --- | --- | --- |
| Idea generation | Group-specific idea-generation activity with each stakeholder group | All 16 stakeholders, organised as family/CYP and healthcare professional groups | 2 |
| Statement review | Follow-up review of refined statements for clarity, meaning and suitability for structuring | Smaller subgroup: 3 family/CYP stakeholders and 3 healthcare professionals | 2 |
| Structuring | Individual sorting and rating of refined statements using Microsoft Word worksheets, organised separately by stakeholder group | All 16 stakeholders | 2 |
| Data analysis | Sorting and rating data analysed using RCMap in R | Research team | Not counted as stakeholder engagement |
| Map interpretation | Group-specific review and discussion of cluster maps, go-zone maps, rating outputs and possible cluster labels | All 16 stakeholders, organised as family/CYP and healthcare professional groups | 2 |
| Consensus | Structured document-mediated consensus activity to compare group outputs and agree final domains and components | All 16 stakeholders | 1 |
| **Total** | **Stakeholder engagement activities** |  | **9** |

**Table 3: Clustered statements (n=27) from healthcare and community stakeholders (n=6) with mean importance and feasibility**

| **Clusters** | **# of statements** | **Statements** | **Mean feasibility** | **Mean importance** |
| --- | --- | --- | --- | --- |
| Clinical and Professional Integration | 7 | 1. named key worker or link practitioner; 2. clear responsibility for follow-up after appointments or discharge; 3. agreed escalation route when needs are not being met; 7. regular multidisciplinary case review; 8. joint discussion across paediatrics, primary care, mental health, school and social care where needed; 10. clear review and escalation arrangements; 19. regular medication reviews with referral back to specialists when needed | 3.67 | 4.71 |
| Accessible and Responsive Care | 8 | 11. neighbourhood or hub-based multidisciplinary working that brings professionals together; 12. care delivered in local, accessible and familiar settings; 13. school-based clinics where appropriate; 14. longer or specialist appointments in primary care; 15. brief staff training on multiple long-term conditions, neurodiversity, disability and cultural responsiveness; 16. prompts for whole-child assessment; 17. reasonable adjustments and flexible appointments; 18. guidance on responding to family context | 3.83 | 4.62 |
| Functional Integration | 5 | 4. shared care plan or hospital passport; 5. standardised letters between hospital, General Practice and community teams; 6. clear documentation of agreed actions; 9. shared action log; 20. a single agreed national medicines formulary for children | 3.60 | 4.50 |
| Whole-Child and Family Support | 4 | 21. routine emotional wellbeing check-ins; 22. brief support within paediatric or neighbourhood pathways; 23. warm handover or referral to appropriate mental health support; 24. respite support for parents and carers | 3.08 | 4.83 |
| Community and System Links | 3 | 25. collection and use of inequality data to target support where it is most needed; 26. child-focused social prescribing link workers and community resources; 27. joint health and housing support for families where housing affects health | 3.72 | 4.50 |

*Note: Mean feasibility and mean importance values are calculated from individual stakeholder ratings for statements within each cluster.*

**Table 4: Clustered statements (n=36) from children, young people and parents/carers (n=10) with mean importance and feasibility**

| **Clusters** | **# of statements** | **Statements** | **Mean feasibility** | **Mean importance** |
| --- | --- | --- | --- | --- |
| Care Coordination | 8 | 3. combined appointment days and video options for suitable less urgent reviews; 4. direct ward access route for known children using a hospital passport; 6. joined-up information-sharing process across community, hospital, school and social care, supported by a multiple long-term conditions summary card for schools and employers; 11. single contact route for family queries; 15. family-held summary of key needs, medicines and contacts; 21. route for parents and carers to submit evidence directly to care assessment panels; 35. transition support from age 16, including a teen care coordinator before transfer to adult care; 36. use of hospital passports at admissions and reviews so staff recognise the child’s needs and preferences | 3.76 | 4.61 |
| Access and Facilities | 12 | 1. safer staffing arrangements for children with intensive complex care needs during hospital admissions; 2. continuing overnight care support during hospital admissions; 5. suitable changing facilities and quiet rooms in hospital and community settings; 12. paediatric outreach clinics in local General Practice hubs; 13. agreed family-initiated or self-referral route when needs change; 14. rapid specialist support route for urgent procedures, including difficult intravenous access; 22. interim care package for children whose needs exceed social care support but do not meet Continuing Health Care eligibility; 26. appointment options compatible with school, nursery and work commitments; 30. diagnostic assessment pathway without additional costs to families or schools; 31. teenager-friendly waiting spaces with sufficient room; 32. accessible General Practice and health centre equipment and facilities, including hoists, plinths and Changing Places toilets; 33. hospital-provided blended diets for children who are enterally fed | 3.92 | 4.66 |
| Information and Support | 12 | 7. professionally translated condition information and clinic letters; 8. simple pathway map showing who to contact and when; 9. text or WhatsApp reminders and follow-up prompts; 10. short culturally tailored print or video information; 16. parent training on condition management and service navigation; 17. practical coaching while waiting for services; 19. help with preparing for appointments and follow-up; 20. support while children are waiting; 23. personalised care process in which professionals understand each child’s needs, problems, symptoms and family context; 24. child voice prompt in consultations and reviews; 27. access to mental health assessment and psychological support; 29. tailored practical support route for single parents | 3.73 | 4.67 |
| School and Community | 4 | 18. signposting to community and peer support; 25. school-parent partnership plan for supporting health needs; 28. school-based health support sessions where appropriate; 34. disability and family representation in decision-making groups | 3.48 | 4.47 |

*Note: Mean feasibility and mean importance values are calculated from individual stakeholder ratings for statements within each cluster.*
